# I’m Getting a Migraine: A Comparative Evaluation of Patient and Clinician Interpretations of Migraine Symptoms

**DOI:** 10.31486/toj.24.0071

**Published:** 2024

**Authors:** Jakob L. Fischer, Anthony M. Tolisano, Alvaro I. Navarro, Waleed M. Abuzeid, Ian M. Humphreys, Nadeem A. Akbar, Sharan Shah, John S. Schneider, Charles A. Riley, Edward D. McCoul

**Affiliations:** ^1^ Department of Otolaryngology – Head and Neck Surgery, Walter Reed National Military Medical Center, Bethesda, MD; ^2^ Department of Surgery, Uniformed Services University of the Health Sciences, Bethesda, MD; ^3^ Department of Otolaryngology – Head and Neck Surgery, Tulane University, New Orleans, LA; ^4^ Division of Rhinology and Endoscopic Skull Base Surgery, Department of Otolaryngology – Head and Neck Surgery, University of Washington, Seattle, WA; ^5^ Division of Rhinology and Skull Base Surgery, Department of Otorhinolaryngology – Head and Neck Surgery, Albert Einstein College of Medicine, Bronx, NY; ^6^ Department of Otolaryngology – Head and Neck Surgery, Washington University School of Medicine, St. Louis, MO; ^7^ The University of Queensland Medical School, Ochsner Clinical School, New Orleans, LA; ^8^ Department of Otorhinolaryngology and Communication Sciences, Ochsner Clinic Foundation, New Orleans, LA

**Keywords:** *Diagnostic errors*, *headache*, *health literacy*, *migraine disorders*, *symptom assessment*, *treatment delay*

## Abstract

**Background:** Patients and providers vary in how they describe common otolaryngology-related complaints. These differences can lead to miscommunication and frustration that may affect patient outcomes and satisfaction. The aim of this cross-sectional survey-based study was to explore the differences in migraine symptom selection by otolaryngology patients and clinicians.

**Methods:** Between June 2020 and October 2022, patients and otolaryngology providers at 5 academic medical centers were asked to select as many symptoms as they felt were related to migraine from a list of 28 common symptom terms in 6 domains: headache-related, eye-related, systemic, sinonasal, facial, and ear-related. The primary study outcome was to assess the differences in patient and clinician perceptions of migraine-related symptoms. A secondary outcome was to assess differences by geographic location.

**Results:** A total of 381 patients and 31 otolaryngology clinicians participated. Patients and providers selected a similar number of symptom terms to define migraine, selecting a median of 10 and 11 symptoms, respectively. Otolaryngology clinicians were more likely than patients to define migraine using eye-related symptoms (difference 10.5%; 95% CI 7.4%, 13.6%) and ear-related symptoms (difference 17.2%; 95% CI 3.4%, 31.0%). Patients were more likely to define migraine using facial symptoms (difference –17.3%; 95% CI –34.1%, –0.5%). Otolaryngologists and patients were equally likely to select headache-related, sinonasal, and systemic symptoms when defining migraine. Minor differences were identified based on geographic location.

**Conclusion:** We found differences between otolaryngologists and their patients in the interpretation of the symptoms of migraine. Clinicians were more likely than patients to describe migraine using eye-related and ear-related symptoms, whereas patients were more likely to describe migraine using facial symptoms. These findings have important counseling and communication implications for clinicians.

## INTRODUCTION

Headaches are a near-universal human experience, with an estimated lifelong prevalence of 96% globally.^1^ The worldwide daily incidence of headache from any etiology is estimated to be as high as 15.8%.^2^ Migraine, specifically, is estimated to have a 12.9% to 15.2% lifetime prevalence,^2^ making it the third most prevalent global disorder.^1^ Migraines are regularly ranked as the second or third highest cause of disability worldwide in both males and females.^3,4^

In 2018, the International Headache Society released the third edition of *The International Classification of Headache Disorders* to further characterize primary and secondary headache disorders and to define clear diagnostic criteria for migraines with and without aura, as well as for a number of migraine subtypes.^4^ Despite these diagnostic criteria and increasing attempts at fully characterizing migraine, diagnosis remains difficult, and delays in diagnosis are common. Up to 53% of patients experience a delay from symptom onset to diagnosis of more than 5 years because of poor patient and clinician recognition^5^ and use of unnecessary diagnostic evaluations.^6^ Migraine and its nonclassic variants also have considerable overlap in symptoms with other conditions such as sinusitis,^7^ Ménière disease,^8^ trigeminal neuralgia, and other secondary headache disorders.^4^ This overlap in symptoms has the potential to create confusion between patients and providers and may be a source of frustration in patient care. Distinguishing among these conditions is critical for the otolaryngologist, as patients with migraines are frequently referred to otolaryngology clinics for management.^9^

Health literacy may also contribute to diagnostic delay. Health literacy is the extent to which patients understand and interpret information provided by their health care professionals. Patients with poor health literacy have shown worse outcomes and higher mortality.^10^ Health literacy can be substantially influenced by receptive communication^11^ and differences in interpretation of medical vernacular. Misunderstandings and barriers to communication may interfere with effective communication. While otolaryngology patients appear to have high levels of health literacy,^12^ the definitions of otolaryngologic complaints such as congestion,^13^ dizziness,^14^ sinus infections,^15^ and reflux^16^ have substantial variability between patients and clinicians.

The primary objective of this study was to investigate potential differences between patients and otolaryngology providers in defining the symptoms related to migraine. Our secondary objective was to determine if any differences in interpretation of symptoms associated with migraine were based on geographic location. We hypothesized that we would find differences between clinicians and patients in their interpretation of the symptoms related to migraine and these differences in interpretation would also vary based on geographic location.

## METHODS

### Study Populations

Participants in this cross-sectional survey-based study were consecutive adult patients (>18 years of age) presenting for routine clinical care at outpatient otolaryngology clinics at academic medical centers in Washington, DC; New Orleans, Louisiana; Seattle, Washington; New York, New York; and St. Louis, Missouri, between June 2020 and October 2022. This study was conducted according to the Strengthening the Reporting of Observational Studies in Epidemiology (STROBE) guidelines for cross-sectional studies.^17^ The following demographic data were collected from the patients who completed the surveys: age, sex, race/ethnicity, and highest level of education.

The provider group was a sample of otolaryngologists and otolaryngology-trained midlevel providers, all of whom were clinicians employed at one of the medical centers participating in this study.

The Walter Reed National Military Medical Center Institutional Review Board approved the study protocol (WRNMMC-EDO-2019-097), and verbal informed consent was obtained from all participants.

### Survey Development and Administration

A list of survey items was compiled from discussions with patients, medical colleagues, and literature review to capture a range of symptoms that may be used to characterize migraines. Items were then selected for inclusion following group discussion facilitated by the senior authors (CAR and EDM). The final survey contained 28 possible symptoms that were randomly arranged into a 7 × 4 grid in the center of a piece of paper to reduce lead-item preference. Participants were asked to circle as many items as they required to answer the question, “What are the symptoms of a migraine?”

The 28 symptoms were grouped into 6 broad domains for the purpose of analysis: headache-related symptoms (headache, throbbing pain, pulsing sensation), eye-related symptoms (eye pain, sensitivity to light, pain behind eyes, flashes of light, aura), systemic symptoms (difficulty concentrating, fatigue, dizziness, imbalance, nausea, vomiting), sinonasal symptoms (thick mucus, loss of smell, blocked or stuffy nose, congestion), facial symptoms (pain in the cheeks, pain in the temples, pain in the forehead, swelling of the face, tightness in the head, pressure in the face, pressure in the forehead), and ear-related symptoms (sensitivity to sound, blocked ears, ear pain).

Patients were asked to complete the survey prior to meeting their health care provider for their appointment to reduce the risk of influencing the results. Patients were instructed to select as many symptoms from the list as they felt were related to migraine, regardless of whether the patient had personally experienced the symptoms.

Clinicians were provided the survey to complete during scheduled departmental academic conferences.

### Statistical Analysis

The surveys were anonymous and contained no personally identifiable information. Item responses were tabulated for each individual symptom and for the 6 domains. A symptom domain was positive if the respondent circled one or more of the domain definitions. The number of clinicians and patients who selected each survey response and the relationships among responses were analyzed. Patients’ and clinicians’ responses were analyzed to identify patterns among symptom domains. Demographic variables of age, sex, race/ethnicity, and highest education level were obtained for the patient population. Univariate analysis of group differences among categorical variables was performed. Confidence intervals were calculated to determine differences in responses between clinician and patient populations. All positive differences in response rates indicate that clinicians were more likely than patients to select a symptom or symptom domain to define migraine. All negative differences in response rates indicate that patients were more likely than clinicians to select a symptom or symptom domain to define migraine. Confidence intervals that exclude zero are statistically significant at a level of *P*≤0.05. A heat map was generated by uploading anonymous matrix data files to Heatmapper^18^ that calculated a pairwise distance matrix using Euclidean measurements.

## RESULTS

A total of 381 patients across 5 different geographic regions (Washington, DC; New Orleans, Louisiana; Seattle, Washington; New York, New York; and St. Louis, Missouri) participated in the study. Two hundred participants (52.5%) were female, and the mean age of all patients was 48.3 ± 16.9 years (Table 1).

**Table 1.** Patient Demographics

		Patients by Geographic Location					
Variable	All Patients, n=381	Washington, DC, n=100	New Orleans, LA, n=101	Seattle, WA, n=58	New York, NY, n=81	St. Louis, MO, n=41	*P* Value
Age, years, mean ± SD	48.3 ± 16.9	42.6 ± 14.5	54.0 ± 16.6	43.6 ± 14.0	48.6 ± 17.9	53.9 ± 17.9	<0.0001
Sex							0.28
Male	175 (45.9)	50 (50.0)	47 (46.5)	29 (50.0)	29 (35.8)	20 (48.8)	
Female	200 (52.5)	50 (50.0)	54 (53.5)	27 (46.6)	51 (63.0)	18 (43.9)	
Race							<0.0001
Caucasian	228 (59.8)	65 (65.0)	71 (70.3)	42 (72.4)	17 (21.0)	33 (80.5)	
African American	90 (23.6)	20 (20.0)	25 (24.8)	3 (5.2)	37 (45.7)	5 (12.2)	
Asian	19 (5.0)	4 (4.0)	2 (2.0)	8 (13.8)	5 (6.2)	0	
American Indian	3 (0.8)	2 (2.0)	1 (1.0)	0	0	0	
Other	32 (8.4)	9 (9.0)	1 (1.0)	3 (5.2)	19 (23.5)	0	
Prefer not to respond	9 (2.4)	0	1 (1.0)	2 (3.4)	3 (3.7)	3 (7.3)	
Ethnicity							<0.0001
Hispanic	56 (14.7)	16 (16.0)	4 (4.0)	4 (6.9)	29 (35.8)	3 (7.3)	
Non-Hispanic	325 (85.3)	84 (84.0)	97 (96.0)	54 (93.1)	52 (64.2)	38 (92.7)	
Highest education level							<0.0001
Elementary school	3 (0.8)	1 (1.0)	0	0	2 (2.5)	0	
High school	69 (18.1)	12 (12.0)	23 (22.8)	4 (6.9)	26 (32.1)	4 (9.8)	
College	181 (47.5)	47 (47.0)	48 (47.5)	24 (41.4)	39 (48.2)	23 (56.1)	
Graduate school	101 (26.5)	40 (40.0)	29 (28.7)	8 (13.8)	13 (16.1)	11 (26.8)	

Note: Data are presented as n (%) unless otherwise indicated.

**Figure 1.**
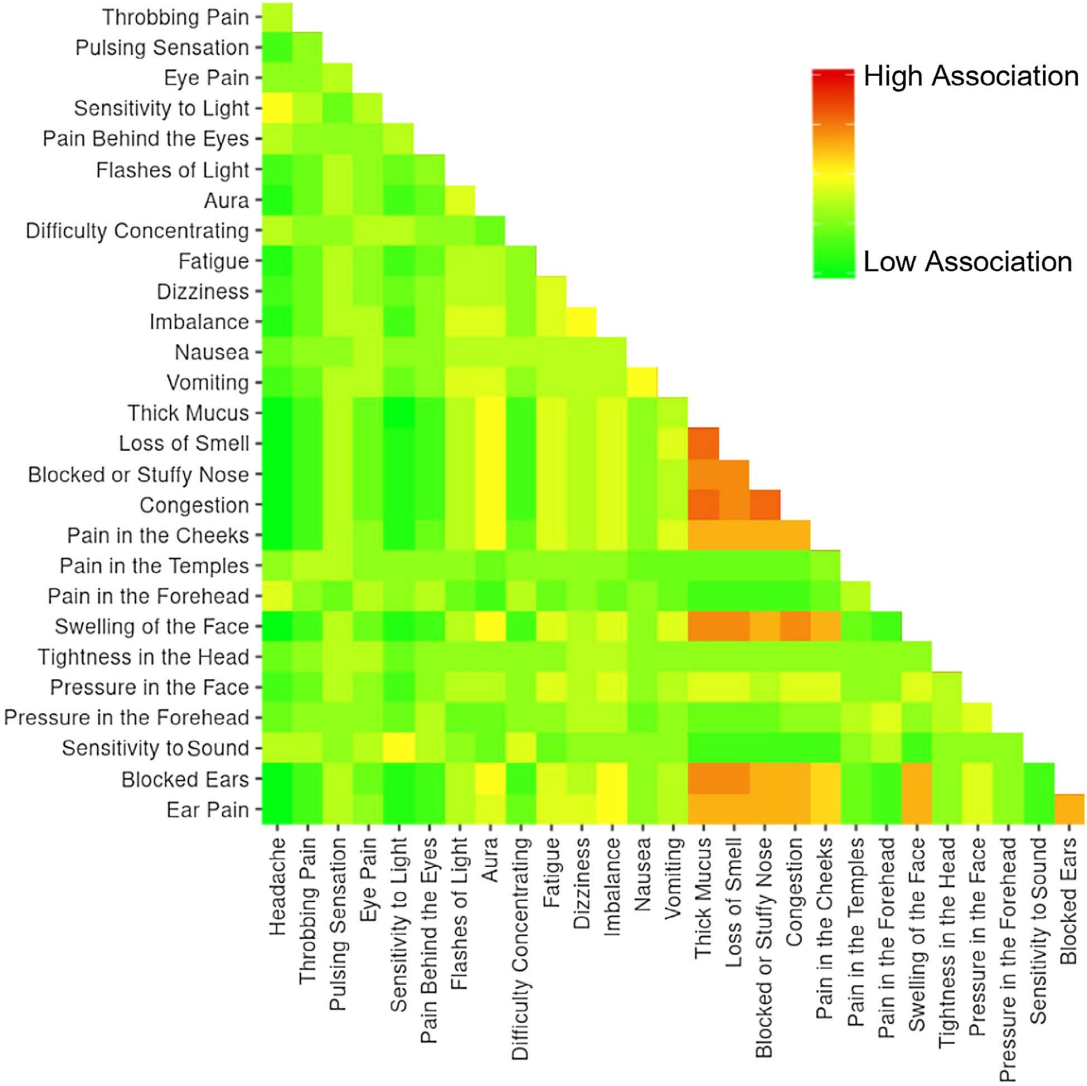
Heat map displaying the associations of individual symptom terms.

Patients most often defined migraine using headache-related symptoms (359, 94.2%), followed by eye-related symptoms (341, 89.5%) and facial symptoms (324, 85.0%) (Table 2). At least 50% of patients associated migraine with the specific symptoms of headache (333, 87.4%), sensitivity to light (295, 77.4%), pain behind eyes (241, 63.3%), throbbing pain (237, 62.2%), sensitivity to sound (237, 62.2%), pain in the forehead (234, 61.4%), difficulty concentrating (226, 59.3%), pain in the temples (208, 54.6%), and eye pain (191, 50.1%). Nearly all patients included symptoms from more than one symptom domain (374, 98.2%), and 300 patients (78.7%) used 4 or more symptom domains to define migraine. Pairwise distance matrix mapping demonstrated few notable associations among individual symptoms. A moderately strong association was seen among the various sinonasal symptoms and between multiple sinonasal symptoms and blocked ears, ear pain, and facial swelling (Figure 1).

**Table 2.** Comparison of Clinician and Patient Selections of the Symptoms Associated With Migraine

				Patient Responses by Geographic Location				
Symptom	Clinicians, n=31	All Patients, n=381	All Patients vs Clinicians Difference (95% CI)^a^	Washington, DC, n=100	New Orleans, LA, n=101	Seattle, WA, n=58	New York, NY, n=81	St. Louis, MO, n=41
**Headache-related symptoms**								
Total	30 (96.8)	359 (94.2)	2.6 (–4.1, 9.2)	93 (93.0)	98 (97.0)	53 (91.4)	76 (93.8)	39 (95.1)
Headache	30 (96.8)	333 (87.4)	9.4 (2.3, 16.4)	87 (87.0)	89 (88.1)	52 (89.7)	69 (85.2)	36 (87.8)
Throbbing pain	20 (64.5)	237 (62.2)	2.3 (–15.2, 19.8)	54 (54.0)	71 (70.3)	38 (65.5)	45 (55.6)	29 (70.7)
Pulsing sensation	15 (48.4)	140 (36.8)	11.6 (–6.6, 29.9)	39 (39.0)	39 (38.6)	18 (31.0)	28 (34.6)	16 (39.0)
**Eye-related symptoms**								
Total	31 (100)	341 (89.5)	10.5 (7.4, 13.6)	85 (85.0)	94 (93.1)	49 (84.5)	73 (90.1)	40 (97.6)
Eye pain	14 (45.2)	191 (50.1)	–5.0 (–23.2, 13.3)	58 (58.0)	49 (48.5)	28 (48.3)	41 (50.6)	15 (36.6)
Sensitivity to light	30 (96.8)	295 (77.4)	19.4 (11.8, 26.9)	73 (73.0)	80 (79.2)	45 (77.6)	59 (72.8)	38 (92.7)
Pain behind eyes	23 (74.2)	241 (63.3)	10.9 (–5.2, 27.1)	67 (67.0)	65 (64.4)	32 (55.2)	51 (63.0)	26 (63.4)
Flashes of light	22 (71.0)	130 (34.1)	36.9 (20.2, 53.5)	29 (29.0)	35 (34.7)	21 (36.2)	25 (30.9)	20 (48.8)
Aura	26 (83.9)	77 (20.2)	63.7 (50.1, 77.2)	16 (16.0)	25 (24.8)	14 (24.1)	11 (13.6)	11 (26.8)
**Systemic symptoms**								
Total	27 (87.1)	307 (80.6)	6.5 (–5.9, 19.0)	76 (76.0)	90 (89.1)	47 (81.0)	59 (72.8)	35 (85.4)
Difficulty concentrating	13 (41.9)	226 (59.3)	–17.4 (–35.4, 0.7)	59 (59.0)	66 (65.4)	33 (56.9)	43 (53.1)	25 (61.0)
Fatigue	5 (16.1)	104 (27.3)	–11.2 (–24.9, 2.5)	30 (30.0)	33 (32.7)	14 (24.1)	18 (22.2)	9 (22.0)
Dizziness	14 (45.2)	129 (33.9)	11.3 (–6.9, 29.5)	35 (35.0)	38 (37.6)	13 (22.4)	31 (38.3)	12 (29.3)
Imbalance	13 (41.9)	104 (27.3)	14.6 (–3.3, 32.6)	27 (27.0)	33 (32.7)	13 (22.4)	24 (29.6)	7 (17.1)
Nausea	24 (77.4)	165 (43.3)	34.1 (18.6, 49.7)	47 (47.0)	48 (47.5)	27 (46.6)	25 (30.9)	18 (43.9)
Vomiting	20 (64.5)	120 (31.5)	33.0 (15.4, 50.5)	24 (24.0)	36 (35.6)	21 (36.2)	24 (29.6)	15 (36.6)
**Sinonasal symptoms**								
Total	6 (19.4)	53 (13.9)	5.4 (–8.9, 19.8)	14 (14.0)	9 (8.9)	6 (10.3)	19 (23.5)	5 (12.2)
Thick mucus	1 (3.2)	18 (4.7)	–1.5 (–8.1, 5.1)	4 (4.0)	5 (5.0)	3 (5.2)	6 (7.4)	0
Loss of smell	3 (9.7)	13 (3.4)	6.3 (–4.3, 16.8)	3 (3.0)	2 (2.0)	1 (1.7)	4 (4.9)	3 (7.3)
Blocked or stuffy nose	4 (12.9)	37 (9.7)	3.2 (–9.0, 12.8)	11 (11.0)	7 (6.9)	3 (5.2)	12 (14.8)	4 (9.8)
Congestion	3 (9.7)	29 (7.6)	2.1 (–8.7, 12.8)	8 (8.0)	5 (5.0)	4 (6.9)	11 (13.6)	1 (2.4)
**Facial symptoms**								
Total	21 (67.7)	324 (85.0)	–17.3 (–34.1, –0.5)	79 (79.0)	91 (90.1)	44 (75.9)	73 (90.1)	37 (90.2)
Pain in the cheeks	6 (19.4)	40 (10.5)	8.9 (–5.4, 23.1)	8 (8.0)	11 (10.9)	3 (5.2)	11 (13.6)	7 (17.1)
Pain in the temples	13 (41.9)	208 (54.6)	–12.7 (–30.7, 5.4)	47 (47.0)	65 (64.4)	26 (44.8)	47 (58.0)	23 (56.1)
Pain in the forehead	20 (64.5)	234 (61.4)	3.1 (–14.4, 20.6)	59 (59.0)	63 (62.4)	32 (55.2)	51 (63.0)	29 (70.7)
Swelling of the face	3 (9.7)	26 (6.8)	2.9 (–7.9, 13.6)	7 (7.0)	9 (8.9)	1 (1.7)	8 (9.9)	1 (2.4)
Tightness in the head	10 (32.3)	172 (45.1)	–12.9 (–30.1, 4.3)	45 (45.0)	49 (48.5)	22 (37.9)	35 (43.2)	21 (51.2)
Pressure in the face	8 (25.8)	120 (31.5)	–5.7 (–21.8, 10.4)	31 (31.0)	32 (31.7)	18 (31.0)	25 (30.9)	14 (34.2)
Pressure in the forehead	10 (32.3)	190 (49.9)	–17.6 (–34.8, –0.4)	49 (49.0)	52 (51.5)	22 (37.9)	49 (60.5)	18 (43.9)
**Ear-related symptoms**								
Total	26 (83.9)	254 (66.7)	17.2 (3.4, 31.0)	66 (66.0)	70 (69.3)	35 (60.3)	53 (65.4)	30 (73.2)
Sensitivity to sound	26 (83.9)	237 (62.2)	21.7 (7.8, 35.5)	60 (60.0)	65 (64.4)	33 (56.9)	50 (61.7)	29 (70.7)
Blocked ears	3 (9.7)	33 (8.7)	1.0 (–9.8, 11.8)	9 (9.0)	11 (10.9)	4 (6.9)	8 (9.9)	1 (2.4)
Ear pain	4 (12.9)	38 (10.0)	2.9 (–9.3, 15.1)	13 (13.0)	8 (7.9)	4 (6.9)	11 (13.6)	2 (4.9)

^a^Values are presented as difference followed by the 95% confidence interval (CI). Positive percent differences indicate that clinicians are more likely to select a symptom to describe migraine, while negative percent differences indicate that patients are more likely to select a symptom to describe migraine. Confidence intervals that do not contain 0 are statistically significant (*P*<0.05).

Note: Data are presented as n (%) except for the All Patients vs Clinician Difference column.

**Figure 2.**
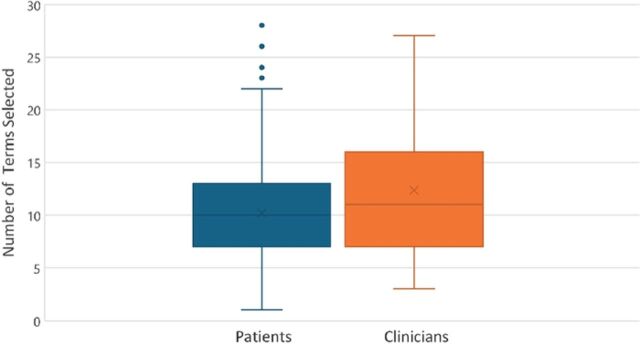
Difference in the number of symptom terms selected by patients and clinicians to describe migraine.

A total of 31 otolaryngology clinicians completed the questionnaire. Otolaryngologists described migraine using a median of 11 symptoms [IQR 7-16] which was comparable to the median of 10 symptoms [IQR 7-13] identified by patients (Figure 2).

**Figure 3.**
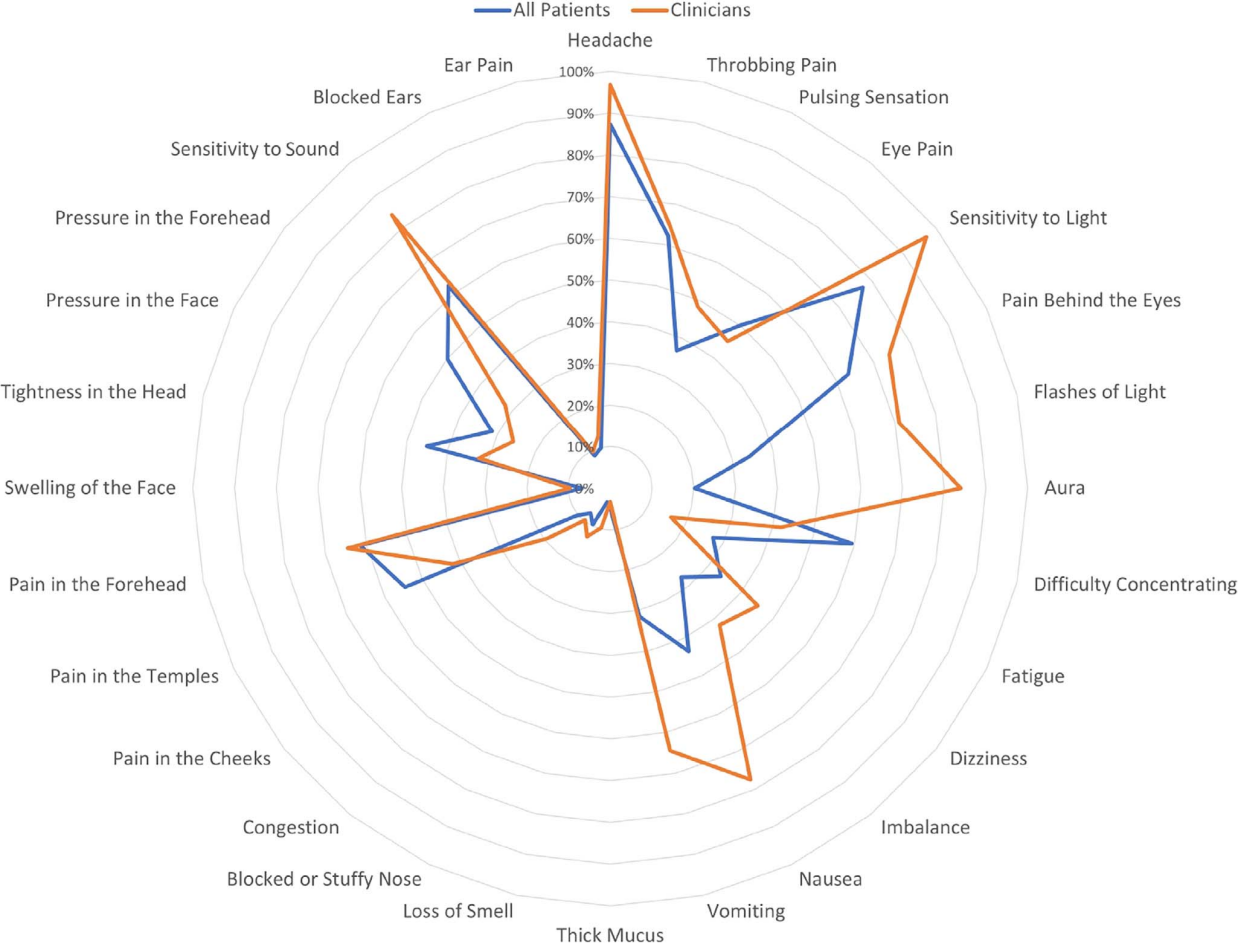
Frequency of individual symptom terms selected by patients and clinicians to describe migraine.

Most providers (28, 90.3%) defined migraine using symptoms from 4 or more symptom domains. Otolaryngologists were more likely to define migraine using symptoms from the eye-related (difference 10.5%; 95% CI 7.4%, 13.6%) and ear-related (difference 17.2%; 95% CI 3.4%, 31.0%) domains. Patients were more likely to select terms related to facial symptoms (difference –17.3%; 95% CI –34.1%, –0.5%). We found no significant difference in the likelihood of either population to define migraine using terms from the headache-related symptoms (difference 2.6%; 95% CI –4.1%, 9.2%), systemic symptoms (difference 6.5%; 95% CI –5.9%, 19.0%), or sinonasal symptoms (difference 5.4%; 95% CI –8.9%, 19.8%) domains. Within the symptom domains for which patients and providers demonstrated no overall difference in response rates (headache-related, systemic, and sinonasal), clinicians were more likely to select 3 individual symptoms to define migraine: nausea (difference 34.1%; 95% CI 18.6%, 49.7%), vomiting (difference 33.0%; 95% CI 15.4%, 50.5%), and headache (difference 9.4%; 95% CI 2.3%, 16.4%) (Table 2). The single largest discrepancy was in the symptom of aura that providers selected 63.7% more often than patients (95% CI 50.1%, 77.2%) (Figure 3).

**Figure 4.**
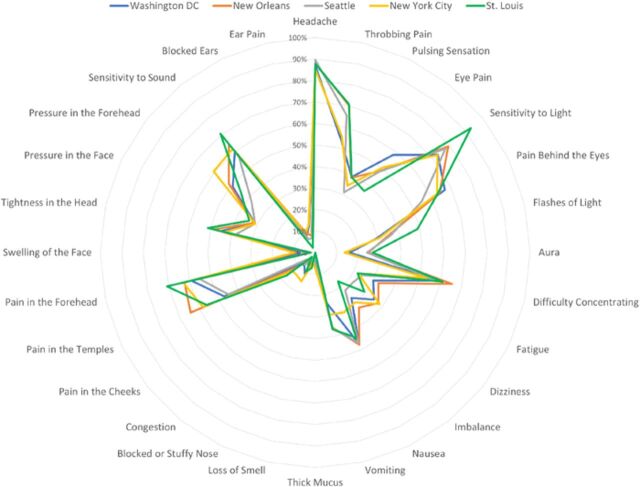
Frequency of individual symptom terms selected by patients to describe migraine according to geographic location.

Geographic location did not have a significant overall impact on patient perception of the symptoms of migraine (Table 3). When comparing clinician and patient responses based on geographic area, we found some notable differences in how patients defined migraine. Patients from New Orleans, Louisiana, were twice as likely to define migraine using facial symptoms than patients in Washington, DC, and were only half as likely to define migraine using eye-related symptoms. Systemic symptoms were more likely to be selected by patients in Washington, DC, and New York, New York, than in New Orleans, Louisiana, and St. Louis, Missouri. In contrast, headache-related, sinonasal, and ear-related symptoms were relatively consistent independent of geographic location (Figure 4).

**Table 3.** Percent Differences Between Patients and Clinicians by Symptom Domains Overall and by Geographic Location

		Patients vs Clinicians Difference by Geographic Location, (95% CI)				
Symptom Domain	All Patients vs Clinicians Difference (95% CI)^a^	Washington, DC	New Orleans, LA	Seattle, WA	New York, NY	St. Louis, MO
Headache-related symptoms	2.6 (–4.1, 9.2)	3.8 (–4.2, 11.8)	–0.3 (–7.3, 6.8)	5.4 (–4.1, 14.9)	3.0 (–5.2, 11.1)	1.7 (–7.4, 10.7)
Eye-related symptoms	10.5 (7.4, 13.6)	15.0 (8.0, 22.0)	6.9 (2.0, 11.9)	15.5 (6.2, 24.8)	9.9 (3.4, 16.4)	2.4 (–2.3, 7.2)
Systemic symptoms	6.5 (–5.9, 19.0)	11.1 (–3.4, 25.6)	–2.0 (–15.3, 11.3)	6.1 (–9.5, 21.6)	14.3 (–1.0, 29.5)	1.7 (–14.3, 17.7)
Sinonasal symptoms	5.4 (–8.9, 19.8)	5.4 (–10.1, 20.8)	10.4 (–4.5, 25.4)	9.0 (–7.0, 25.0)	–4.1 (–20.8, 12.6)	7.2 (–10.0, 24.3)
Facial symptoms	–17.3 (–34.1, –0.5)	–11.3 (–29.6, 7.0)	–22.4 (–39.8, –4.9)	–8.1 (–27.9, 11.7)	–22.4 (–40.1, –4.7)	–22.5 (–3.7, –41.3)
Ear symptoms	17.2 (3.4, 31.0)	17.9 (1.9, 33.8)	14.6 (–1.2, 30.3)	23.5 (5.5, 41.6)	18.4 (1.9, 35.0)	10.7 (–8.1, 29.5)

^a^Values are presented as difference followed by the 95% confidence interval (CI). Positive percent differences indicate that clinicians are more likely to select a symptom domain to describe migraine, while negative percent differences indicate that patients are more likely to select a symptom domain to describe migraine. Confidence intervals that do not contain 0 are statistically significant (*P*<0.05).

## DISCUSSION

To identify potential barriers to communication, this study evaluated the differences between patients and providers who were asked to define the symptoms associated with migraine. We found that patients and providers varied in their interpretation of the term migraine but found no significant differences in patient interpretation based on geographic location. Overall, patients and providers used a similar number of terms to define migraine, but the focus of those symptoms varied significantly. Providers were more likely to define migraine using terms from the eye-related and ear-related symptoms domains, whereas patients were more likely to define migraine using symptoms in the facial symptoms domain.

Semantic differences may exist for several reasons, such as education level, exposure to medical care, and insurance status, but overall, these differences highlight reasons communication may be limited^19^ and can negatively impact the patient-provider relationship.^20^ Because the diagnosis of migraine relies heavily on patient reporting of subjective symptoms, many patients experience substantial delays in the diagnosis and treatment of migraine.^5^ This delay in diagnosis may be in part attributable to these differences in interpretation of the symptoms associated with migraine.

When specifically examining the symptoms that clinicians were more likely to select than patients, we found differences in interpretation among the symptoms classically associated with migraine with aura: sensitivity to light, flashes of light, sensitivity to sound, and the term aura itself. Non-aura–related symptoms that were more commonly selected by providers vs patients were headache, nausea, and vomiting.

Part of this difference in interpretation among symptoms may be directly related to differences in personal experience related to migraine and portrayal of migraine by popular media and advertising. While migraine has an estimated 15% lifetime incidence among the global population,^2^ the lifetime incidence of migraine with aura is reported to be much lower at 6% to 8%.^21,22^ Migraine with aura predominately affects women, with a female:male ratio of 3:2 and first migraine often beginning at menarche or early in the fourth decade of life, in contrast with migraine without aura that maintains a normal age distribution.^22^ A likelihood among our patient population is that a minority of patients may have experienced migraine with aura and therefore may be less likely to associate those symptoms with migraine. Interestingly, while a minority of patients (77, 20.2%) selected the term aura while defining migraine, the majority of our patient population identified some of the individual symptoms related to aura, specifically sensitivity to light (295, 77.4%) and sensitivity to sound (237, 62.2%), albeit at a lower rate than their clinician counterparts. These responses indicate that patients are aware of the symptoms associated with aura but may not be aware of the specific encompassing medical term. These findings have implications in patient education and screening tools, as asking patients if they experience aura relating to migraine may be inadequate to fully elucidate the symptoms they may be experiencing.

While migraine is a common complaint, the majority of patients in this study may not have personally experienced a migraine, and their interpretation may be influenced by symptoms seen in popular media, advertising, or retellings from social contacts. Gvantseladze et al searched the web for migraine images and found that the majority of the 283 images they examined depicted migraine sufferers as adult White women with their eyes closed and their hands on their temples, foreheads, midface, or eyes.^23^ Despite these images being common representations of migraine in media, they may create an unrealistic depiction of migraine. Raffaelli et al presented frequently used stock photos depicting a migraine to 367 migraine patients and 331 health care workers and found that fewer than half of the participants found these images to be realistic depictions of migraine, and the majority of patients were unable to identify their own migraine experience in the photos.^24^ These findings indicate a disparity between the advertised interpretation of how a migraine patient appears and the reality of the symptoms patients may experience. Our patient population may reflect these differences as well. In our patient population, the facial symptoms domain was the only domain that patients were more likely to select than providers and includes symptoms such as pain and pressure in the forehead and temples that are commonly depicted in search engine results and the media, although the majority of these individual symptoms did not reach statistical significance.

Understanding migraine and being able to communicate with patients regarding the symptoms associated with migraine is critically important to the otolaryngologist. Schreiber et al examined 2,991 patients with a history of “sinus headache” and found that 88% of patients presenting for evaluation of sinus-related symptoms were experiencing migraines.^25^ A separate study found that 81.5% of migraine patients were misdiagnosed as having sinusitis, leading to an average delay in diagnosis of 7.8 years.^7^ Among these patients, 87.7% received medical treatment and 12.3% received surgical treatment for sinus disease, but the treatment was unsuccessful in relieving their sinus symptoms in 84.9% and 76.9% of patients, respectively, before they received a diagnosis of migraine.^7^ Similarly, patients presenting with dizziness and vertigo should be screened for vestibular migraine to differentiate migraine from conditions such as Ménière disease.^8^ Van Ombergen et al estimated that up to 16% of patients presenting to an otolaryngology dizziness clinic may have vestibular migraine and could benefit from appropriate prophylactic treatment.^26^

### Limitations

This study design has inherent limitations. Patient-response surveys are subject to misinterpretation, miscomprehension, response bias, limited participation, and hidden agendas associated with completion of paperwork. Our survey was not subjected to validation testing. Five medical treatment facilities representing a geographically diverse population were included, but some geographic regions had comparatively few responses which may limit the ability to detect statistically meaningful differences in patient populations. This study only explored the symptoms associated with the word migraine among patients presenting to an otolaryngology clinic independent of their chief complaint. Further study would be required to explore the interpretation of these symptoms with primary care physicians and neurology providers where the symptoms associated with migraine may vary significantly. Moreover, we do not know which patients had experienced or presented with migraine-related symptoms, which may aid in further refinement of the symptoms associated with migraine. This study included midlevel providers and physicians under the umbrella of clinicians. These groups may have potential differences in the interpretation of the symptoms related to migraine that were not accounted for in this study. Future studies for investigation include surveying patients with and without a history of migraine to determine differences in interpretation among these populations, as well as inclusion of providers in other fields of medicine such as neurology, primary care, and emergency medicine.

## CONCLUSION

This survey-based study found differences in the interpretation of the symptoms relating to migraine between otolaryngologists and their patients. Otolaryngologists were more likely to define migraine using eye-related and ear-related symptoms, while patients were more likely to define migraine with facial symptoms. These findings have important implications in patient education and symptom screening for the otolaryngologist given the high prevalence of migraine.

## Notes

*This article meets the Accreditation Council for Graduate Medical Education and the American Board of Medical Specialties Maintenance of Certification competencies for Patient Care and Medical Knowledge.*
